# Pain management in people with inflammatory arthritis: British Society for Rheumatology guideline scope

**DOI:** 10.1093/rap/rkae128

**Published:** 2024-11-19

**Authors:** Ian C Scott, Opeyemi Babatunde, Christopher Barker, Rebecca Beesley, Richard Beesley, Hollie Birkinshaw, Mel Brooke, Hema Chaplin, Lara Chapman, Coziana Ciurtin, James Dale, Dervil Dockrell, Emma Dures, Kathyrn Harrison, Meghna Jani, Charlotte Lee, Maura McCarron, Christian D Mallen, Assie O’Connor, Claire Pidgeon, Tamar Pincus, Dee Pratt, Yeliz Prior, Karim Raza, Zoe Rutter-Locher, Seema Sharma, Katie Shaw, Samantha Small, Tilli Smith, Lesley Tiffin, Jordan Tsigarides, Mikalena Xenophontos, Nicholas G Shenker

**Affiliations:** Primary Care Centre Versus Arthritis, School of Medicine, Keele University, Staffordshire, UK; Haywood Academic Rheumatology Centre, Midlands Partnership University NHS Foundation Trust, Staffordshire, UK; Primary Care Centre Versus Arthritis, School of Medicine, Keele University, Staffordshire, UK; Community Pain Management Service, Mersey Care NHS Foundation Trust, Mersey, UK; Juvenile Arthritis Research (JAR), Tonbridge, UK; Juvenile Arthritis Research (JAR), Tonbridge, UK; School of Primary Care, Population Sciences and Medical Education, Primary Care Research Centre, University of Southampton, Southampton, UK; Expert by experience, Bath, UK; Health Psychology Section, Institute of Psychiatry, Psychology and Neuroscience, King’s College London, London, UK; School of Health and Society, Centre for Applied Health Research, University of Salford, Salford, UK; Leeds Institute of Rheumatic and Musculoskeletal Medicine, University of Leeds, Leeds, UK; Division of Medicine, Centre for Adolescent Rheumatology, University College London, London, UK; Department of Rheumatology, NHS Lanarkshire, Lanarkshire, UK; Bone Research Group, University of Edinburgh and NHS Lothian, Edinburgh, UK; School of Health and Social Wellbeing, University of the West of England (UWE), Bristol, UK; Department of Paediatric Rheumatology, Birmingham Children’s Hospital, Birmingham, UK; Centre for Epidemiology Versus Arthritis, Centre for Musculoskeletal Research, The University of Manchester, Manchester, UK; NIHR Manchester Biomedical Research Centre, Manchester University NHS Foundation Trust, Manchester Academic Health Science Centre, Manchester, UK; School of Psychology, University of Southampton, Southampton, UK; Department of Rheumatology, Belfast Health and Social Care Trust, Belfast, UK; School of Psychology, Queen’s University Belfast, Belfast, UK; Primary Care Centre Versus Arthritis, School of Medicine, Keele University, Staffordshire, UK; Haywood Academic Rheumatology Centre, Midlands Partnership University NHS Foundation Trust, Staffordshire, UK; Department of Paediatric Occupational Therapy, Birmingham Women’s and Children’s NHS Foundation Trust, Birmingham, UK; School of Psychology, University of Southampton, Southampton, UK; Department of Musculoskeletal Physiotherapy, Surrey Downs Health and Care Community Services, Epsom and St Helier University Hospitals NHS Trust, Surrey, UK; Centre for Human Movement and Rehabilitation, School of Health and Society, University of Salford, Salford, UK; Department of Rheumatology, Hywel Dda University Health Board, Wales, UK; Rheumatology Research Group, Institute of Inflammation and Ageing, University of Birmingham, Birmingham, UK; Department of Rheumatology, Guy’s and St Thomas’ NHS Trust, London, UK; Department of Rheumatology, Manchester University NHS Foundation Trust, Manchester, UK; Department of Physiotherapy, Bolton NHS Foundation Trust, Bolton, UK; Department of Paediatric Rheumatology, Southampton Children’s Hospital, Southampton, UK; Primary Care Centre Versus Arthritis, School of Medicine, Keele University, Staffordshire, UK; Department of Rheumatology, Newcastle Hospitals NHS Foundation Trust, Newcastle, UK; Department of Rheumatology, Norfolk and Norwich Hospital, Norwich, UK; Norwich Medical School, University of East Anglia, Norwich, UK; Kennedy Institute of Rheumatology, University of Oxford, Oxford, UK; Department of Rheumatology, Oxford University Hospitals, Oxford, UK; Department of Rheumatology, Cambridge University Hospitals, Cambridge, UK; Department of Medicine, University of Cambridge, Cambridge, UK

**Keywords:** inflammatory arthritis, rheumatoid arthritis, psoriatic arthritis, axial spondyloarthritis, juvenile idiopathic arthritis, pain, analgesic, pharmacological, non-pharmacological

## Abstract

Pain is a common symptom in people with inflammatory arthritis (IA), which has far-reaching impacts on their lives. Recent electronic health record studies demonstrate that UK-based pain care in people with IA commonly involves the prescribing of long-term opioids and gabapentinoids, despite an absence of trial evidence for their efficacy. Patient surveys suggest that non-pharmacological pain management is underused. A UK-specific guideline on pain management for people with IA is required to resolve this. This scoping document outlines the context and prioritized clinical questions for the first British Society for Rheumatology (BSR) guideline on pain management for people with IA. The guideline aims to provide evidence-based recommendations on how pain can be best managed in people with IA (including its assessment, and pharmacological and non-pharmacological treatments), ensuring that people with IA in the UK are offered evidence-based pain management strategies. The guideline is for healthcare professionals involved in the care of people with IA of all ages and genders, people with IA and their families and carers, NHS managers and healthcare commissioners, and other relevant stakeholders such as patient organizations. It will be developed using the methods outlined in the BSR’s ‘Creating Clinical Guidelines’ protocol.

## Why the guideline is needed

Pain is a common problem for people with inflammatory arthritis (IA), with many experiencing daily pain and reporting dissatisfaction with their arthritis pain [1, 2]. UK-based electronic health record studies indicate that pain care in people with IA commonly involves the prescribing of long-term opioids and gabapentinoids [3, 4], despite an absence of evidence for efficacy [5] and potential adverse events [6, 7]. UK-based patient surveys also suggest that non-drug IA pain care (such as physiotherapy-supported exercise regimens and orthoses) are underused [8, 9]. A guideline from the British Society for Rheumatology (BSR) on pain management in people with IA is needed to improve pain and its care in people with IA. By providing recommendations developed through multi-stakeholder involvement (including healthcare professionals who care for people with IA and people with IA) based on the best available evidence, it will support the provision of evidence-based pain management to people with IA treated in the UK.

## Key facts and figures

Recent studies using national electronic health record data from primary care show that IA is common in adults, with over 1% of people aged ≥18 years, and over 2.5% of those aged >65 years meeting criteria for a diagnosis of rheumatoid arthritis (RA), psoriatic arthritis (PsA) or axial spondyloarthritis (AxSpA) in England in 2020 [10]. Juvenile idiopathic arthritis (JIA) is rarer, with <0.1% of children and young people having a validated primary care recorded diagnosis of JIA in 2018 [11], although the condition can continue into adulthood [12]. Optimizing pain care in people with IA will therefore improve the lives of a substantial number of people across the UK.

Current rheumatology care for adults with IA focuses on delivering ‘treat-to-target’ strategies, in which disease activity is regularly measured [e.g. using the Disease Activity Score for 28 Joints (DAS28) in those with RA] and disease-modifying anti-rheumatic drugs (DMARDs) escalated until the target of remission or low disease activity is achieved. In children and young people treat-to-target approaches are also used, although they are less well defined and trial evidence comparing this approach with another or no strategy are lacking [13]. Treat-to-target has transformed many outcomes in people with IA, leading to lower disability levels, less radiological damage and improved quality of life [14, 15]. However, while it also improves pain in people with IA [16, 17], it does not fully control it, even in those achieving these targets [18]. The limitations of DMARDs for pain are shown in observational studies, including in the BSR Biologics Registry (in which 79% of people with RA receiving biologic DMARDs belonged to a ‘persistent pain’ trajectory [19]) and clinical trials [20]. While cohorts of people with early RA show lower disease activity levels and better physical quality of life in the treat-to-target era, pain levels remain similar in pre- and post-2002 cohorts [21].

Longitudinal studies show that many people with IA suffer from persistent pain, including those receiving high-cost biologic DMARDs [19, 22–24]. The impact of pain is far-reaching, being the dominant predictor of psychosocial health [25], independently predicting work disability [26], and being associated with worse quality of life, functioning, mental health, fatigue and well-being [27–30]. Consequently, people with IA consistently rate pain and improved pain care as a key priority [31–33]. Pain management in people with IA is complicated by its multidimensional and multifactorial nature, with nociceptive pain (from actual/threatened damage to non-neural tissue and nociceptor activation e.g. from synovitis), neuropathic pain (from a lesion/disease of the somatosensory nervous system) and nociplastic pain (from altered nociception in the absence of tissue damage/inflammation or a somatosensory nervous system lesion/disease), all playing contributing roles [23, 34, 35]. Nociplastic pain is particularly common, with a systematic literature review and meta-analysis of the prevalence of fibromyalgia in people with RA, PsA and ankylosing spondylitis (a subtype of AxSpA) reporting a pooled prevalence of 21%, 18% and 13%, respectively [35].

## Current practice

Recent UK-based electronic health record studies demonstrate that pain care for people with IA focuses on the prescribing of analgesics [3, 4, 36]. For example, in one study examining the annual prevalence of analgesic prescriptions in the Clinical Practice Research Datalink Aurum (a large database of routinely collected data from >1400 general practitioner (GP) practices spanning 20% of England), the annual prevalences of analgesic, non-steroidal anti-inflammatory drug (NSAID), opioid and gabapentinoid prescriptions in 2020 were 64.5, 22.3, 39.0 and 9.9 per 100 person-years, respectively [3]. This is despite limited trial evidence of efficacy (except for NSAIDs in AxSpA) [5, 37, 38], but many potential harms (including fractures and overdose with opioids and upper gastrointestinal complications with NSAIDs) [6, 39]. Published data on the extent to which non-drug therapies are used for IA pain management in the UK are limited, but suggest they are underused. In a 2009 survey of 1400 people with RA in England 44%, 40%, 28% and 13% reported having received NHS-based physiotherapy, occupational therapy, podiatry and orthoses, respectively [9]. A more recent survey (in 2022) of people with AxSpA reported that, among 294 people diagnosed in the previous 5 years, only 40% stated they had receiving information about physical exercises tailored specifically to their AxSpA in the 12-months post-diagnosis [8]. In the whole sample of 913 people, while 82% rated ‘advice on how to manage pain levels’ as ‘very important’, only 39% rated their experience of this as ‘very/quite positive’.

## Equity considerations

Health Survey for England 2017 data demonstrate that, in the general population, people are more likely to experience chronic pain (pain that persists or recurs for more than 3 months) if they live in deprived communities, are from minoritized ethnic backgrounds, are female, have more than two long-term conditions or are older [40]. Similarly, in Scottish Health Survey 2022 data, the proportions of people with chronic pain were higher in females, older people and in those living in more deprived areas [41]. There is also evidence that in the general population, opioid use generally [42], and in people with chronic non-cancer pain long-term opioid use specifically [43], is commoner in more deprived areas. These factors are also likely to be important in people with IA, with the annual prevalence of opioid or gabapentinoid prescriptions higher in people with IA living in England that are older, female or living in areas of deprivation and North England [3]. More recently, in 2023, the Arthritis and Musculoskeletal Alliance conducted a national inquiry into musculoskeletal health inequalities, concluding that in patients with inflammatory rheumatic conditions (such as IA), low socioeconomic status is associated with increased pain and poorer outcomes, for which there is no biological basis [44].

## Previous guidance

While many contemporary guidelines exist for the management of IA, these primarily focus on reducing disease activity with DMARDs, providing little guidance on pain. For example, in National Institute for Health and Care Excellence (NICE) RA guidelines, the few pain-specific recommendations are that short-term NSAIDs and hand exercises can be considered, periodic multidisciplinary team assessments should occur, people should have access to specialist physiotherapy to learn about transcutaneous electrical nerve stimulators and wax baths for short-term pain relief, and early surgical reviews should be offered for persistent pain from joint damage [45]. This is because these guidelines have focused on pain related to disease activity and are targeted at secondary care services. Similar approaches have been used in NICE guidelines related to PsA and other types of peripheral spondyloarthritis (SpA) [46]. Only one contemporary guideline (from the European Alliance of Associations for Rheumatology [EULAR]) primarily addresses pain management in people with IA [47]. This guideline does not consider JIA. While providing useful information for clinical practice, it is based on a relatively historic umbrella review (considering systematic reviews until 2015), with many systematic reviews published since. It also does not consider pharmacological treatments in detail (with its underpinning umbrella review evaluating non-drug care only) and combines recommendations for both IA and osteoarthritis (whose models of care differ). The 2021 NICE guideline on chronic pain provides general advice on pain management for chronic primary pain (in which no underlying condition adequately accounts for the pain or its impact) [48]. When chronic primary and secondary pain co-exist (which can often occur in people with IA), it recommends using clinical judgement to inform shared decision-making about the management options in the relevant NICE guideline. As NICE guidelines for IA do not focus on pain, healthcare professionals and people with IA may consequently feel uncertain as to the best way to manage chronic pain in this condition.

## What the guideline will cover

### Who are the target users of the guideline?

The guideline is for all healthcare professionals involved in the management of people with IA, people with IA and their families and carers, NHS managers and healthcare commissioners, and other stakeholders such as patient organizations.

### Which population will the guideline apply to?

It will cover pain management in adults, children and young people with IA [including RA, PsA and other forms of peripheral SpA, AxSpA, JIA and undifferentiated IA]. All ages and genders will be considered.

### Settings

The guideline will be of relevance to all UK healthcare settings (primary/community, secondary and tertiary care).

### Areas that will be covered

The definition of pain from the International Association for the Study of Pain will be used, which defines it as ‘an unpleasant sensory and emotional experience associated with, or resembling that associated with, actual or potential tissue damage’ [49]. The guideline will consider literature in three key areas relevant to pain in people with IA: (1) the assessment of pain; (2) pharmacological interventions for pain; and (3) non-pharmacological interventions for pain. It will consider evidence for all types of pain (nociceptive, neuropathic and nociplastic) [50].

### Areas that will not be covered

The guideline will not consider the following areas: (1) acute pain due to clearly defined non-rheumatological issues (e.g. infection, post-operative pain, trauma); (2) pain-management procedures (e.g. surgical interventions, vagal nerve stimulation or intra-articular steroids); (3) public health interventions for pain; (4) pain management in people with rheumatological conditions where IA can occur, but is not traditionally considered to fall within the umbrella-term of ‘IA’ [such as crystal arthritis, connective tissue diseases (including systemic lupus erythematosus), vasculitis and autoinflammatory syndromes]—although the findings from this guideline are likely to be relevant to people with these conditions; and (5) foot pain (being addressed in BSR’s ‘management of foot health in people with inflammatory arthritis’ guideline) [51]. It will also not cover non-pain aspects of IA care, which are addressed in other national guidelines from NICE and BSR.

## Key issues and clinical questions

The following clinical questions have been identified by the Guideline Working Group to address within the guideline. Where relevant, these have been framed in the Population, Intervention, Comparator and Outcome format.

### Assessment of pain

In people with IA (population):

- Which pain factors (e.g. sensitization) and pain-related factors (e.g. mood) should be considered when assessing pain?
- Which interpersonal consultation-based factors should be considered when assessing pain?
- Which outcome measures should be used when assessing pain?
- Should pain assessments be in-person or remote?
- How often should pain assessments take place?

### Pharmacological treatments for pain

In people with IA (population), which of the following pharmacological treatments (intervention) relative to placebo or other pharmacological/non-pharmacological treatments (comparator), improve pain (primary outcome) and what are their effects on quality of life, function, stiffness, adverse events, analgesic use, sleep, fatigue, mental health, education and employment (secondary outcomes):

- Analgesics—paracetamol, oral NSAIDs, topical NSAIDs, nefopam, opioids.
- Neuromodulators—anti-depressants, gabapentinoids, topical capsaicin, cannabinoids.
- Immunosuppressants—systemic glucocorticoids, conventional synthetic (cs)DMARDs, biologic (b)DMARDs and targeted synthetic (ts)DMARDs.

### Non-pharmacological treatments for pain

In people with IA (population), which of the following non-pharmacological treatments (intervention) relative to placebo or other non-pharmacological/pharmacological treatments (comparator), improve pain (primary outcome) and what are their effects on quality of life, function, stiffness, adverse events, analgesic use, sleep, fatigue, mental health, education and employment (secondary outcomes):

- Exercise and physical activity.
- Psychological therapies.
- Ergonomics.
- Orthotics (excluding orthoses for foot pain, which are considered in the BSR management of foot health in people with IA guideline).
- Other: education; weight management and diet; sleep and fatigue management; digital technologies; complementary therapies; medical devices; and support from others.

## Service organization and delivery within NHS England and devolved nations

People with IA require holistic pain care (reflecting the multidimensional and multifactorial nature of pain) and may present with pain-related symptoms in all healthcare settings (including primary care, where practitioners may feel less confident in assessing disease activity). Collaborative working across healthcare boundaries, and ensuring equitable access to MDT services, is therefore crucial to providing evidence-based, equitable pain management. Recent decades have seen an erosion in the rheumatology MDT, with few UK rheumatology departments having a full complement of healthcare professionals, and the composition and staffing levels of the rheumatology MDT varying substantially by geographical location [52]. Consequently, the Guideline Working Group will provide commissioner-friendly dissemination materials, engage with Specialist Societies and Royal Colleges in guideline implementation and develop relevant audit and quality improvement tools to measure and improve pain care across the healthcare system.

## Guideline working group

The multi-disciplinary Guideline Working Group comprises the following members from across the UK: Ian C. Scott (co-chair, Rheumatologist); Nicholas G. Shenker (co-chair, Rheumatologist); Opeyemi Babatunde (Evidence Synthesis Researcher); Christopher Barker (General Practitioner); Rebecca Beesley (patient and charity representative); Richard Beesley (carer and charity representative); Hollie Birkinshaw (Research Psychologist); Mel Brooke (patient representative); Hema Chaplin (Chartered Psychologist); Lara Chapman (Podiatrist); Coziana Ciurtin (Adolescent and Adult Rheumatologist and BSR Standards, Audit, and Guidelines Working Group liaison); James Dale (Rheumatologist); Dervil Dockrell (Occupational Therapist); Emma Dures (Chartered Psychologist); Kathyrn Harrison (Paediatric Rheumatologist); Meghna Jani (Rheumatologist); Charlotte Lee (Research Psychologist); Maura McCarron (Rheumatology Specialty Doctor); Christian Mallen (General Practitioner); Assie O’Connor (Rheumatology Pharmacist); Claire Pidgeon (Paediatric Occupational Therapist); Tamar Pincus (Chartered Psychologist); Dee Pratt (Physiotherapist); Yeliz Prior (Occupational Therapist); Karim Raza (Rheumatologist); Zoe Rutter-Locher (Rheumatology Specialist Registrar); Seema Sharma (Rheumatology Specialist Registrar); Katie Shaw (Physiotherapist); Samantha Small (Paediatric Rheumatology Nurse Specialist); Tilli Smith (Evidence Synthesis Researcher); Lesley Tiffin (Nurse Practitioner); Jordan Tsigarides (Rheumatology Specialist Registrar); and Mikalena Xenophontos (Rheumatology Specialist Registrar).

## Dissemination

The guideline will be accompanied by infographic and video summaries in both patient-friendly and commissioner-friendly formats. The Guideline Working Group will engage with Specialist Societies, Royal Colleges and patient associations to disseminate the guideline to their target audiences. Following publication, the Guideline Working Group will seek to activate local champions within the healthcare community, including BSR’s Pain Special Interest Group, to optimize guideline uptake, facilitated by the guideline’s audit and quality improvement tools. The guideline is expected to be published in 2026.

## Data Availability

No new data were generated or analysed in support of this work.
